# The Impact of Zinc Oxide Nanoparticles on Male (In)Fertility

**DOI:** 10.3390/ma13040849

**Published:** 2020-02-13

**Authors:** Ana Rita Pinho, Sandra Rebelo, Maria de Lourdes Pereira

**Affiliations:** 1 Department of Medical Sciences, University of Aveiro, 3810-193 Aveiro, Portugal; arapinho@ua.pt; 2 Neuroscience and Signalling Laboratory, Institute of Biomedicine (iBiMED), 3810-193 Aveiro, Portugal; 3 CICECO-Aveiro Institute of Materials, University of Aveiro, 3810-193 Aveiro, Portugal

**Keywords:** ZnO nanoparticles, spermatogenesis, biomedicine, cytotoxicity, ROS, apoptosis

## Abstract

Zinc oxide nanoparticles (ZnO NPs) are among nanoscale materials, attracting increasing attention owing to their exceptional set of characteristics, which makes these engineered nanoparticles a great option for improving the quality and effectiveness of diagnosis and treatment. The capacity of ZnO NPs to induce reactive oxygen species (ROS) production, DNA damage, and apoptosis represents a promise for their use in both cancer therapy and microbial treatment. However, their intrinsic toxicity together with their easy entrance and accumulation in organism have raised some concerns regarding the biomedical use of these NPs. Several studies have reported that ZnO NPs might induce cytotoxic effects on the male reproductive system, compromising male fertility. Despite some advances in this area, the knowledge of the effects of ZnO NPs on male fertility is still scarce. Overall, a brief outline of the major ZnO NPs biomedical applications and promises in terms of diagnostic and therapeutic use will also be explored. Further, this review intends to discuss the effect of ZnO NPs exposure on the male reproductive system and speculate their effects on male (in)fertility.

## 1. Introduction

Currently, nanoparticles (NPs) are defined as solid colloidal particles with less than 100 nm length/width in at least one dimension [1,2,3]. At the beginning of the 21st century, the interest in nanotechnology has emerged. Nanotechnology was defined as the study, manipulation, and control of matter at the nanometre scale according to its future application [2,3,4]. Interestingly, NPs are classified according to the material type used, dimension, morphology, composition, uniformity, agglomeration, and origin process. On the basis of the type of material used, NPs can be divided into four categories: metallic nanoparticles (e.g., Au, Ag, Cu, Fe, Zn NPs), metal and non-metal oxides (e.g., FeO, VO, AlO, ZnO NPs), semiconductor nanoparticles (e.g., ZnS, CdSe, ZnSe, CdS NPs), and carbon-based NPs. Regarding the origin process, NPs are classified as natural or engineered. Engineered nanomaterials are produced by mechanical abrasion, engine exhaust, and smoke, or are synthesized by physical, chemical, or biological methods [1,3,5,6]. NPs have been synthesized in a wide variety of nanostructures.

Recently, there is growing interest in the metal oxide nanoparticles, like ZnO NPs, owing to their unique set of characteristics currently used for different approaches, from biomedicine to industry and agriculture. Zinc oxide can be referred as a multifunctional material owing to its unique physical and chemical properties. It is important to note that zinc ion (Zn^2+^) is the most essential microelement found in all body tissues. Zn^2+^ is located intracellularly, being indispensable for metalloproteins’ function, providing a unique platform for protein subdomains to interact with either DNA or other proteins (zinc finger motifs). These biological properties made the ZnO NPs an excellent option when compared with other metal nanoparticles, in part owing to its natural biocompatibility. Besides that, Zn^2+^ is an active element, and simultaneously a strong reducing agent that easily oxidizes, generating ZnO. In fact, under normal conditions, the conversion of Zn^2+^ in ZnO is unidirectional. However, changes in pH and thermal conditions may induce the conversion of ZnO in Zn^2+^ [7]. Additionally, recent studies mention that the toxicity induced by ZnO NPs is because of the dissolution of ZnO NPs in Zn^2+^, altering the Zn^2+^ homeostasis, which is an important feature of the potential toxicity [8,9,10]. These nanoparticles present several advantages: High transparency, low toxicity, good size, good light trapping properties, natural abundancy, photoluminescence, good photocatalysis, inexpensive, good semiconductor, high catalytic organic transformation, chemical sensing, bound to several transition metal oxide nanoparticles, stable under UV radiation, and capable of generating reactive oxygen species (ROS) [5,11].

Nanobiotechnology has developed quickly and there is a huge variety of nanoparticles applications and preparation processes. However, this “nano-expansion” should be closely monitored as small amounts of nanoparticles are associated with high levels of toxicity, which could have an impact on environmental, animal, and human health. The broad application of ZnO NPs from the agriculture field to the textile industry makes humans constantly vulnerable to their exposure. Additionally, the development of biomedical applications, for example, in drug delivery systems, imaging, molecular diagnostics, and cancer therapy, has also raised some questions about ZnO NPs biosafety [5,11,12,13], and several studies addressed these issues.

Spermatogenesis is a complex process that originates highly specialized male reproductive cells. It comprises several regulated stages, from proliferation and differentiation of spermatogonia, meiosis, and spermiogenesis [14]. The spermatogonia cells suffer several mitotic divisions to both self-renew and produce undifferentiated diploid spermatogonia. Upon several rounds of differentiation and mitosis, spermatogonia originates the spermatocytes, which in turn suffers two meiotic divisions, and during the second spermatogenic phase, round haploid spermatids are produced. These spermatids are subjected to dramatical morphological alterations throughout the spermiogenesis, originating the elongated spermatids and finally the mature spermatozoa [15,16]. Recently, novel insights about the biological relevance of nuclear envelope proteins for mammalian spermatogenesis and male fertility were reviewed [17].

However, the ZnO NPs possible adverse effects on the male reproductive system are to date not well understood. Few studies have proposed ZnO NPs as a cytotoxic inducer in both testis and male germ cells, in a dose and time of exposure dependent manner. The aim of this review is to summarize the scientific contributions of the impact of ZnO nanoparticles for male reproductive cells’ function, providing the discussion of these results and presenting future perspectives for the study of male (in) fertility.

## 2. ZnO Nanoparticles, A Variety of Biomedical Applications

Currently, there is a huge variety of ZnO NPs applications, from agriculture to the electronic industry and biomedical area. The conformational state of ZnO NPs means this type of nanoparticle is very promising and useful. There are several studies addressing the better NPs’ conformation to answer the 21st century necessities.

**Figure 1:**
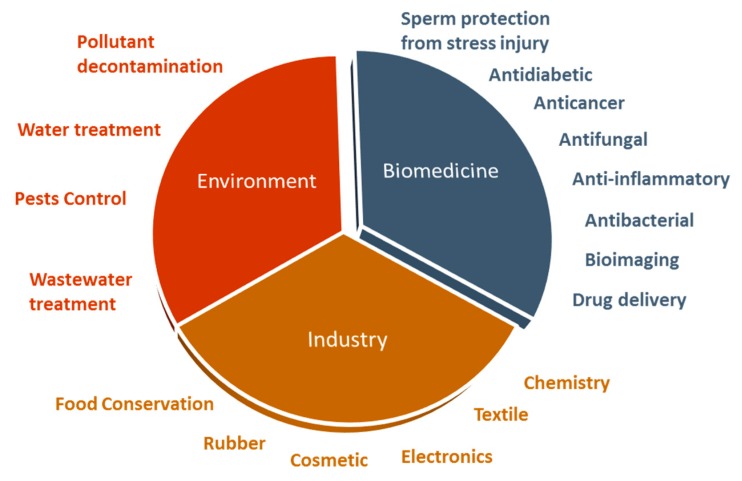
The ZnO nanoparticles, with a variety of applications in biomedicine, industry, and environment.

The exceptional properties of ZnO NPs make them an excellent biomedical agent, being able to generate reactive oxygen species (ROS) and induce apoptosis, when used at high concentrations [18]. However, these NPs might be used as antioxidant agents at lower concentrations [19]. These are considered important characteristics for antimicrobial, anticancer, anti-inflammatory, and antidiabetic activities proposed for these NPs. Additionally, ZnO NPs can be used as a sperm protective agent from cryopreservation and diabetes injuries. Further, ZnO NPs have been successfully exploited as delivery systems of therapeutic agents and also as a bioimaging agent [11,12] (Figure 1).

Although, is important to note that the effects of ZnO NPs depend on the size, the concentration used, the morphology, the synthesis process, the surface area, the cell type tested [20,21,22], and the organism type (only in the case of bacteria and fungi) [5,23,24,25,26]. Small sizes, higher concentrations, and high frequency of administration doses enhance its effects [5,19,25,26].

ZnO NPs can be used as an antibacterial nanomaterial given their inherent properties, like high specific surface area and a high capacity to block a wide range of pathogenic agents [12]. ZnO NPs prevent bacteria adhesion, spreading, and breeding in medical devices, and are very useful in medical applications like pharmaceutical or cosmetic industries, as well as for textile modifications [12,27].

The main antibacterial toxicity mechanisms of ZnO NPs are based on their ability to increase ROS generation, especially when exposed to light [12,24,28,29,30], causing peroxidation of the lipid membrane, leading to its dysfunction and consequent rupture [23,29]. Furthermore, the electrostatic interactions of ZnO NPs with the bacteria surface were revealed to be a possible antibacterial mechanism that caused membrane damage, especially with high ZnO NPs’ concentrations [31,32]. These membrane alterations result from blockage of transport channels by ZnO NPs that causes starvation and eventually cell death [23]. Also, the antibacterial activity might involve the accumulation of ZnO NPs in the outer membrane or in the cytoplasm of bacterial cells triggering Zn^2+^ release, and thus bacterial cell membrane disintegration, active transport inhibition, membrane protein damage, and consequent genomic instability and alterations on membrane permeability [12,23,33,34,35].

Additionally, ZnO NPs are also considered antifungal agents, making them suitable for the food safety and agriculture industries [5,25,36,37]. Like in bacterial cells, the increased ROS production is the mainly cause of *Candida albicans* cell death and ZnO NPs’ exposure to blue light enhances the oxidative stress [25]. A different study has shown that, besides ZnO NPs increasing the ROS levels, the growth inhibition verified in *Botrytis cinerea* and *Penicillium expansum* results from alterations on fungi morphology that are NPs’ concentration-dependent [38]. Additionally, antifungal studies will be necessary to improve the potential applications of ZnO NPs as an antifungal agent, as the ZnO NPs’ effects on yeast, for example, are still very scarce.

Besides antimicrobial activity, ZnO NPs could have potential anticancer properties and are considered a new alternative to cancer chemotherapy and radiotherapy. They are able to target multiple cancer cell types and simultaneously perform several key functions, including inhibiting cancer proliferation, drug-resistant cancer sensitization, preventing cancer recurrence and metastasizing, and reactivating cancer immunosurveillance [39]. ZnO NPs could be a selective anti-cancer agent, inducing higher ROS production in cancer cells when compared with normal cells and, together with the increased sensitivity for cancer cells, result in selective cell death of these tumoral cells [5,40]. These metal oxide NPs are able to alter the antioxidant mechanisms of cancer cells [18], leading to the activation of intracellular apoptosis signalling pathways [41] and consequent cell cycle arrest, preventing cell damage propagation to the daughter cells. [42]. Moreover, ZnO NPs exhibit a strong preferential ability to kill dividing cancer cells relatively to quiescent cells of the same lineage, suggesting that the mechanisms of ZnO NPs’ toxicity might be associated with the proliferative potential of the cells. This inherent differential toxicity raises exciting opportunities for NPs as anticancer agents. The selectivity of these nanomaterials can be improved by changing the NP design towards adding tumor targeting ligands to tumor-associated proteins, or by using NPs for drug delivery. These observations may provide the basis for the development of new rational strategies to protect against NPs toxicity or enhance the destruction of cancer cells [43].

Besides the use of ZnO NPs as anticancer activity, the loading of anticancer drugs into ZnO NPs presents some advantages that solve serious limitations of common drug carriers, such as enhancing the drug circulation for considerable periods of time, maintaining the relevant therapeutic concentrations, and facilitating the drug adsorption [44]. NPs are small enough to pass through the capillaries, to target specific sites of cancer cells, and also to allow a controlled release of the drug, reducing the overall amount of drug used and minimizing undesirable side effects. The innate anticancer activity of ZnO NPs combined with the therapeutic activity of the drug loaded contributes to a more effective drug cancer treatment. Furthermore, it provides better targeting of the highly toxic chemotherapeutic drugs as well as a controlled release of the drug, and showed low toxicity towards normal cells, producing very few side effects [12,36,39,45,46]. ZnO NPs might be also useful for DNA transfer, for real time imaging of gene transfer, for targeted gene delivery and gene silencing, and also for next-generation cancer applications [45,47].

Several studies have indicated that ZnO NPs have different mechanisms for inflammation inhibition that are very useful in autoimmune [43] and inflammatory diseases and in drug designing and targeting, as well as in the food and cosmetic industry. They may also offer a plausible solution for cancer and various types of inflammation treatment using, for instance, UV rays with minimal side-effects [39,48,49,50]. Interesting studies regarding atopic dermatitis revealed that ZnO NPs treatment decreases local skin inflammation on a mouse model with atopic dermatitis [51] and patients with atopic dermatitis [52], as a result of the anti-inflammatory properties of ZnO NPs [51,52], as well as high antioxidative and anti-bacterial capacities [52].

ZnO NPs might be used as a promising antidiabetic agent and diabetes complications reducer [12,53]. ZnO NPs effectively reverse diabetes-induced pancreatic structural, ultrastructural, and functional injuries [19,54]; normalize blood glucose [19,54] and serum insulin levels [53,54]; and restore the sensitivity of the insulin receptor to insulin [53]. This is explained because ZnO NPs might be an antioxidant agent at lower doses [19] and zinc acts directly on the insulin signalling pathway [12,55], improving hepatic glycogenesis.

Besides the role in insulin metabolism, the lower doses of ZnO NPs might have a protective effect on diabetes rat sperm, owing to their antioxidant properties. Sperm from diabetic rats is poor in number and sperm cells present a weak motility. Additionally, the serum testosterone levels are decreased. However, in the presence of ZnO NPs alone or in combination with insulin, an improvement in the quality of sperm and an increase of testosterone production is observed [56]. Moreover, ZnO NPs supplementation to nicotine exposed rats minimizes the harmful effects caused by exposure, through decreasing oxidative stress and increasing expression of steroidogenic enzymes, improving male fertility [57]. Also, ZnO NPs lower levels can protect the male reproductive system from damage induced by anticancer [58] and antibacterial drugs [59].

Furthermore, the use of ZnO NPs in sperm cryopreservation medium has been proven to be a protective factor to sperm, preventing common cell damage in cryopreserved sperm, namely DNA damage and cell membrane lipid peroxidation. Moreover, the acrosomal reaction is not altered in cells treated with ZnO NPs relative to control, meaning that this type of nanoparticles does not affect the cell fertility, increasing the motility of sperm compared with cryopreserved sperm [60]. However, the sperm underlying the protective mechanism is not well known and the majority of studies reported the ZnO NPs as a cytotoxic factor, as will be reviewed below.

## 3. ZnO Nanoparticles: Route of Exposure and Accumulation in Organism

Despite nanotechnology being a contemporary science, human exposure to nanoparticles has occurred throughout human history and dramatically increased during the industrial revolution [4]. Of note, when the industrial emissions do not fulfil the current environmental and public health safety standards, human exposition to NPs is inevitable and uncontrolled. Currently, despite that the industrial emissions standards are regulated, the number of NPs applications significantly increased, as described below. Furthermore, new NPs commercial applications have been developed, involving new properties, leading to new biological interactions and unexpected toxicity [61].

Owing to their small size, NPs have the ability to penetrate the skin, the lungs, the gastrointestinal tract, and also the blood brain barrier (BBB) [62]. These tissues, except the BBB, are in constant contact with the environment, facilitating the NP entrance. Additionally, injections and implants are other possible routes of exposure. Thus, nanoparticles can translocate into the circulatory and lymphatic systems, and ultimately to body tissues and cells, though interaction with subcellular structures [1].

ZnO NPs are a type of nanomaterial with a huge demand in biomedicine, industry, and agriculture, among other areas, meaning that the human exposure to these NPs is high, and like the generality of NPs, they can invade the human organism tissues and cells. ZnO NPs have been described as an easily accumulated nanomaterial, whose accumulation rate differs depending on the tissue type. Liver, kidney, lung, brain, and spleen are the organs with high ZnO NPs accumulation levels [63], presenting signals of cytotoxicity as a consequence of that exposure [64,65,66,67]. Besides that, metal nanoparticles have the capacity to cross the blood–testis barrier (BTB), in part by its size but also by the production of an inflammatory response that compromises the BTB integrity [62]. BTB controls the adluminal environment in which germ cells develop by influencing its chemical composition, so changes on BTB represent a risk to spermatogenesis [68,69]. It is possible to speculate that ZnO NPs may also cross the BTB, inducing testicular toxicity. Given the importance of this issue, this should be addressed in future studies. However, the knowledge on how ZnO NPs cross BTB is indispensable for assessing this toxic mechanism in the male reproductive system.

## 4. ZnO Nanoparticles and their Effects on Male Reproductive System—Analysis of In Vitro and In Vivo Studies

As described above, the variety of ZnO NPs applications is wide. Considering that human exposure to this type of metallic nanoparticle is high, it is relevant and essential to evaluate the effects of the ZnO NPs at several levels, including the histological, cellular, and molecular levels. Some studies have focused on the effects of ZnO NPs on spermatogenesis and have suggested that ZnO NPs have an impact on the male reproductive system. Tables 1 and 2 display representative studies, summarizing the main contributions of in vitro and in vivo studies, respectively.

### 4.1. In Vitro Studies

Only three in vitro studies were performed to evaluate the cytotoxic effects of ZnO NPs on the male reproductive system (Table 1). In vitro analyses of ZnO NPs effects on spermatogenesis, particularly on male germ cells, are scarce and mainly focused on cytotoxicity evaluation in different cell types. From all over the stages of spermatogenesis, the cytotoxicity of ZnO NPs was just analysed at very few cell stages, namely at the spermatocytes and spermatozoa, which indicates that their effect on male germ cells and consequently the reproductive system is not well established and deserves further investigation. In addition, the ZnO NPs effects on regulatory and supportive cells, Sertoli and Leydig cells, were also analysed and discussed. All the studies were performed using mouse cell lines, except one that was carried on human sperm samples. Viability, ROS production levels, DNA damage, and apoptosis levels were the parameters evaluated in these in vitro studies (Table 1).

**Figure 2:**
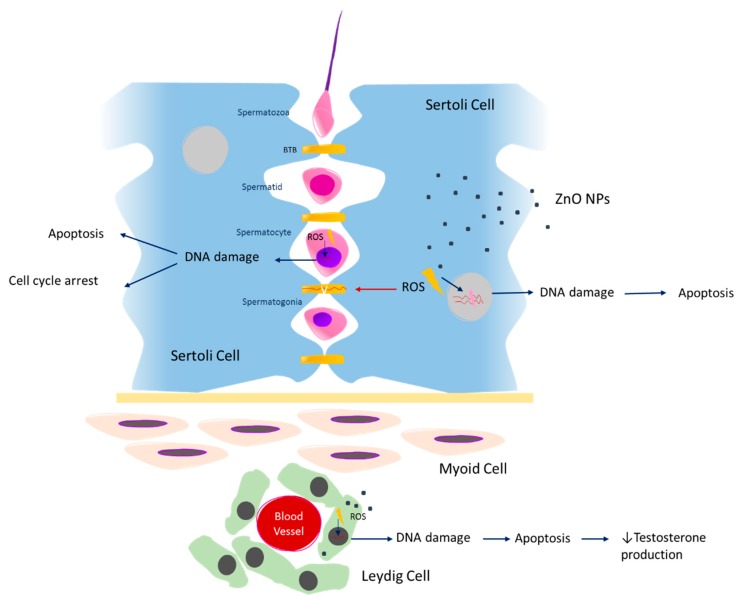
The cytotoxic effects of ZnO NPs on spermatogenesis. BTB—blood-testis barrier; ROS—reactive oxygen species; DNA—deoxyribonucleic acid; ZnO NPs—zinc oxide nanoparticles.

As mentioned above, the BTB is a crucial component for normal spermatogenesis function. Previous studies evaluated the expression of tight junction proteins in Sertoli cells, namely claudin-5, occludin, ZO-1, and connexin-43, which promotes the adhesion between Sertoli cells forming the BTB [70]. As a consequence of ZnO NPs exposure, increased ROS production is observed and the expression of these BTB proteins is decreased [70,71] (Figure 2). The authors proposed that increased ROS production levels compromise BTB through down-regulating of tight junction proteins in Sertoli cells, leading to DNA damage and cell cycle arrest at the S-phase (Table 1). Further, it was also proposed that ROS possibly activates the Erk1/2 proteins, inducing an increase of tumor necrosis factor alpha (TNF-α) cytokine levels, leading to alterations in tight junction proteins [72,73]. Therefore, it is possible to deduce that the integrity of BTB is altered in the presence of ZnO NPs, compromising the essential conditions for spermatogenesis progression [70]. To date, it is not known how the ZnO NPs cross the BTB, and this issue should definitely be explored in future studies.

Another interesting study defined the concentration of 1000 μg/mL of ZnO NPs as the cytotoxic dose for human sperm cells, given that a significant reduction in cell viability is observed. Additionally, the increase of cell mortality was observed 180 minutes after NPs exposure [74]. According to the results in Table 1, the cytotoxicity of ZnO NPs on the male reproductive system depends on concentration, time of exposure, and also cell type [70,71,74]. However, the structural characteristics of ZnO NPs, for example, size and surface area, were not considered as a variable of analysis. The cytotoxic effects of ZnO NPs were also evaluated using mouse spermatocytes [70]. The authors described an increase in DNA damage as well as cell cycle alterations. An increased expression in cell cycle proteins and an increased number of cells in S-phase and a decrease in the G1 and G2 phase. These alterations were reduced by the use of an antioxidant, indicating that oxidative stress plays a role in ZnO NPs cell cycle arrest [70] (Figure 2).

From all in vitro studies described, only two evaluated apoptosis in Leydig [71,75] and Sertoli cells after exposure to ZnO NPs [71]. These authors found that the apoptosis is associated with ROS formation, and with loss of mitochondrial membrane potential, which leads to the increase of apoptosis in Sertoli and Leydig cells associated with nuclear DNA damage [71,75] (Figure 2). These results clearly indicate a possible negative effect of ZnO NPs on spermatogenesis progression, as the number of Sertoli and Leydig apoptotic cells increases after exposure to these nanoparticles. In mouse Leydig cells, autophagy stimulated by oxidative stress might protect cells from ZnO NPs-induced apoptosis [75].

### 4.2. In Vivo Studies

Several animal models and routes of administration were used to study the in vivo consequences of ZnO NPs exposure on spermatogenesis. These in vivo studies evaluated the impact of ZnO NPs on the male reproductive system using different animal models (Table 2). This type of investigation is important to evaluate the histological and biochemical alterations observed in testis when exposed to ZnO NPs.

Most of these studies were performed in rodents using several techniques, including histological and biochemical analysis. In general, the parameters studied included testicular histology, seminiferous tubule (ST) diameter and epithelium height, oxidative stress, apoptosis, and genotoxicity markers. Most studies characterized ZnO NPs, although in some studies, that information was not available.

#### 4.2.1. In Vivo Studies in Mammalian Animal Models

Most of the in vivo studies reported in Table 2 evaluate changes in testicular and epididymal tissues after exposure of rats or mice to different ZnO NPs concentrations. The histological pattern was similar in both rats or mice, with the formation of multinucleated giant cells [76,77], disorganization of germ cells layers, detachment and sloughing of immature germ cells, and vacuolization of the epithelium of ST after exposure to a high concentrations of ZnO NPs [75,76,77,78,80,86]. These histological alterations are indicative of functional damage in Sertoli cells, which are responsible for the support and protection of germ cells during spermatogenesis [76]. 

Spermatogenesis arrest has been described as a consequence of exposure to high ZnO NPs concentrations [75,76,77,78,80]. The appearance of immature germinal cells in the epididymis [77], as well as degenerated and desquamated spermatocytes [77,78] and sperm cells [77] in the STs lumen and epididymis, respectively, is evident. In addition, the number of germinative cells is reduced [75,76,77,78,86] and the STs of Wistar Han rats were almost empty of spermatids and spermatozoa after exposure to 400 mg/kg of ZnO NPs. The number of Leydig cells, an important cell responsible for testosterone production, also decreased in exposed animals [87,88]. These results are consistent with data obtained from in vitro study using a Leydig cell line, which reports a decrease in Leydig cells’ viability and an increase of apoptosis after in vitro exposure to ZnO NPs [71] (Figure 2). Furthermore, according to a recent study, the testis toxicity after exposure to ZnO NPs occurs via apoptosis and the ER-stress signaling pathway, activated by ROS [86].

Additionally, analysing the morphology of seminiferous tubules, a decrease in the diameter and height of the epithelium resulting from germ cell loss is notable, owing to the apoptotic effect of ZnO NPs on spermatogenic cells [71,76,77,83]. In addition to the testis and epididymis, other studies analysed histological changes in seminal vesicles and prostate [80], with an increase in their weight [84]. The occurrence of inflammation in the dilated area of prostate acini and the hyperplasia of epithelial lining cells of prostatic acini was detected in mice and rats exposed to ZnO NPs [80,85]. In seminal vesicles, the changes were also significative, with the detection of mononuclear cells infiltrating the stroma and the appearance of mild to moderate proliferation of epithelial cells [80]. According to these data, exposure to ZnO NPs has repercussions on all male reproductive systems in a dose-dependent manner.

Regarding the sperm quantity and quality, ZnO NPs cause harmful effects, reducing the number and motility of sperm cells and increasing the number of morphological abnormalities such as double head, small head, formless head, and double tail [64,71,76,78,81,84]. A significant reduction in sperm viability has also been observed in several studies [64,78,81]. Interestingly, Hussein et al., 2016 revealed that these changes in sperm quality could be alleviated by co-exposure with quercetin, a potent antioxidant. The number of Leydig cells also decreases after exposure to a low concentration of ZnO NPs, and as a consequence, serum testosterone levels decrease, as reported in Wistar Han rats [78] and Kunming mice [75,86]. Additionally, the same authors explored the influence of ZnO NPs on steroidogenesis enzymes by assessing the mRNA levels of steroidogenic proteins in testis samples, namely 3β-hydroxysteroid dehydrogenase (3β-HSD), 17β-hydroxysteroid dehydrogenases (17β-HSD), and steroidogenic factor 1 (Nr5A1) [78]. These are enzymes responsible for the conversion of pregnenolone, a cholesterol derivative, into progesterone and androstenedione, a progesterone derivative, in testosterone, respectively [87]. Nr5A1 is a transcription factor that regulates the expression of several steroidogenic enzymatic genes on Leydig cells (such as 3β-HSD and aromatase) and is involved in the steroidogenesis pathway and synthesis of testosterone [88]. Moreover, a recent study reported a significant down-regulation of steroidogenic acute regulatory protein (StAR) in testis, very important in testosterone synthesis, responsible for the transport of hydrophobic low-density cholesterol from the cytosol to the mitochondrial inner membrane [86,89]. The expression of mRNA steroidogenic proteins showed a significant decrease in ZnO NPs-exposed groups, which justifies the lowering in serological testosterone levels [78,86].

As mentioned in previous studies, ZnO NPs are a source of oxidative stress owing to their accumulation on liver. In order to evaluate antioxidant parameters in rats and mice, some studies analysed the activity of superoxide dismutase (SOD), glutathione peroxidase (GPx), reduced glutathione (GSH), catalase (CAT), and total antioxidant capacity (TAC). The levels of lipid peroxidation marker malondialdehyde (MDA) and of total oxidant status (TOS) were also assessed to evaluate the oxidative stress. A significant reduction was found in the activity of antioxidant enzymes (CAT, SOD, GPx) and of GSH [78], just as TAC was decreased in the blood sample of rats [64,85]. TOS and the lipid peroxidation level have been shown to increase significantly after exposure to a high concentration of ZnO NPs [64,78,85]. Thus, ZnO NPs may be considered an inhibitor of the antioxidant machinery and an oxidative stress inducer. A reduction in sperm viability occurs as a result of ROS formation in liver. ROS induce chromosomal alterations in blood samples and DNA damage, indicating that rats exposed to a high concentration of ZnO NPs have higher levels of ROS in organism that induce apoptosis of the sperm cell [81].

Contrary to the majority of studies, sheep exposure to ZnO NPs improved the sperm parameters and neutralized the effects of ROS by increasing its antioxidant activity. These results are attributed to the zinc antioxidant activity and its role in the stabilization of sperm according to the study. According to the authors, it is important to note that different animal species have differential nutritional requirements for spermatogenesis, and until now, there are no in vivo published studies about the effect of ZnO NPs on spermatogenesis reported in any other animal model than rodents. In addition, the dose of ZnO NPs is lower compared with other studies. Thus, future studies are needed to clarify these results, repeating the study with higher doses of exposure [82].

#### 4.2.2. In Vivo Studies in Non-Mammalian Animal Models

Non-mammalian in vivo studies have similar results to those obtained from mammalian in vivo studies. In testis from Japanese quail treated with ZnO NPs, the morphological analysis of ST revealed a decrease in diameter and height of the epithelium, as observed in mammalian in vivo studies resulting from germ cell loss, and an apoptotic effect of ZnO NPs on spermatogenic cells. Further, the serum testosterone levels decreased with exposure to ZnO NPs. In addition to the histological analysis of the Japanese quail testis, egg hatchability was also evaluated after sperm exposure to different concentrations of ZnO NPs, to assess the sperm functionality. The cytotoxic effects of ZnO NPs in sperm cells have repercussions in fertility, causing infertility and a higher incidence of embryonic deaths, reducing hatchability rates and inducing teratogenic effects on their embryos [83]. These results emphasize the importance of toxicity study in male germ cells, once ZnO NPs are capable to reduce the fertility from Japanese quail.

The effect of ZnO NPs exposure on germ cell death was also evaluated in *C. elegans*. Exposure of *C. elegans* to ZnO NPs caused an increase in the apoptotic cell number, resulting from a change in apoptotic gene regulation. The apoptotic genes *cep-1*, *cep-13*, *efl-2*, *egl-1*, *lin-35*, and *sir-2.1* were significantly upregulated in the presence of ZnO NPs, promoting cell apoptosis [79]. Although it is necessary to be aware that this animal model is hermaphrodite, in this study, no distinction was made between germ cells. Thus, it is necessary to study the expression of upregulated genes in male mammalian cells to better understand the apoptosis mechanism behind ZnO NPs in germ cells.

## 5. Conclusions and Future Perspectives

The applications of ZnO NPs in biomedicine are numerous, given the multiple advantages conferred by the physicochemical properties of these nanomaterials. However, these nanoparticles induce significative cytotoxic effects on spermatogenesis. These cytotoxic effects are dose and time of exposure dependent, indicating that a high concentration and a high time of exposure induce more toxicity. Toxicity is normally induced by increased ROS production and by inhibition of antioxidant cell response, inducing DNA damage, with cell cycle arrest culminating in apoptosis of male germ cells. The same occurs with Sertoli and Leydig cells, whose role of support and regulation of spermatogenesis is well known (Figure 2).

Oxidative stress also has an important harmful effect on Sertoli cells besides apoptosis, as the tight junctions suffer significative effects that might compromise the spermatogenesis progression (Figure 2). Histological analysis revealed sloughing and denaturation of male germ cells and disorganization of germ cells layers as a consequence of ZnO NPs exposure. Regarding Leydig cells, the histological analysis indicated a decrease of their number as a consequence of the rat’s exposure to ZnO NPs. The serum levels of testosterone also decreased, which also compromises the spermatogenesis progression, which is testosterone-dependent. These reductions were explained by the decreased expression of steroidogenic proteins in testes samples, the decreased mitochondrial membrane potential, and the increased apoptosis in Leydig cells.

The study of ZnO NPs effects on male fertility is still scarce, mainly at the in vitro and in vivo levels. It is important to extend the cytotoxic evaluation of ZnO NPs to the first cell stage of spermatogenesis, spermatogonia, and later stages to understand whether ZnO NPs are capable of compromising all spermatogenesis or whether their harmful effects are reversible. Further, the sperm fertilization capacity is an important parameter that should be determined in mammalian exposed to ZnO NPs, in order to understand their direct effect in male fertility. In future, it is important to address cytotoxicity and histotoxicity studies in human samples, given the scarcity of such an approach. 

Besides that, it will be interesting to investigate ways to control the toxic effects of ZnO NPs reported so far. Several studies have developed new forms of conjugating ZnO NPs with other materials that reduced natural cytotoxicity. The toxicity of these new nanocomplexes also needs to be investigated in testicular cells. In addition, it is important to note that all studies evaluate ZnO NPs toxicity based on concentration and exposure time, but the effects of any NP also depend on their physicochemical properties. Thus, future studies should be conducted to assess their effects on the male reproductive system based on size, surface area, and method of synthesis.

## Figures and Tables

**Table 1:** In Vitro studies of ZnO nanoparticles (NPs) effects on male germ cells. Abbreviations: DNA, deoxyribonucleic acid; BTB, blood–testis barrier; DMEM, Dulbecco′s modified Eagle′s medium; GPx, glutathione peroxidase; GSH, reduced glutathione; MDA, malondialdehyde; RPMI, Roswell Park Memorial Institute medium; ROS, reactive oxygen species; MMP, mitochondrial membrane potential; ERK ½: extracellular signal-regulated kinase 1/2; TNF-α, tumor necrosis factor alpha; Bcl-2, B cell lymphoma-2; Bax, Bcl-2-associated X protein; LC3, microtubule-associated protein 1A/1B-light chain 3; Atg5, autophagy related 5.

ZnO NPs’ Characteristics	Objective	Cell Type	ZnO NPs Concentration (μg/mL)	Parameters	Results	Reference
**Size:** 50 nm **Shape:** amorphous	Evaluate the cytotoxicity of ZnO NPs on viability of spermatozoa	Spermatozoa (Human)	10, 100, 500, 1000	—Viability	—The toxicity depends on concentration and time of exposure; —Higher concentrations and higher exposure periods induce higher toxicity	[74]
**Size:** 70 nm **Shape:** spherical **Nature:** crystalline **Dispersion:** polydisperse and agglomerate in quasi-spherical and hexagonal structures **Surface roughness:** high (22.9 nm)	Investigate the toxicity of ZnO NPs in testicular cells	Leydig cell Sertoli cell (Mouse)	0, 5, 10, 15, 20	—Cellular uptake of ZnO NPs —Viability —MMP and ROS levels —Apoptosis and DNA Damage	—ZnO NP aggregates in the cytoplasm and in nucleus; —The toxicity depends on the concentration (≥10 μg/ml) and the exposure time (≥6 h), and not on Zn^2+^ release; —ROS production increase, leading to loss of MMP originating apoptosis of Leydig and Sertoli cells and DNA damage	[71]
**Size:** 177 nm **Shape:** spheroid or ellipsoid **Zeta Potential:** −27.4 ± 1.0 mV **Purity:** >97%	Explore the effects of sublethal doses of ZnO NPs and their underlying mechanisms on male germ cells	Sertoli cell Spermatocyte (Mouse)	0, 0.04, 0.08, 0.4, 0.8, 4, 8, and 16	—Viability —ROS, GSH, and MDA levels; —Permeability, MMP, and cytochrome C —BTB junction proteins levels; —Erk1/2 and TNF-α levels; —DNA damage and cell cycle;	—The sublethal dose of ZnO NP is 8 µg/mL; —GSH levels decrease and MDA levels increase; —Sertoli cell membrane disruption and cellular invasion; —ROS levels production increase compromising BTB, by down-regulating the expression of BTB junction proteins, causing DNA damage and cell cycle arrest at S-phase at spermatocytes	[70]
**Size:** 30 nm; **HS:** 66.36 ± 0.39 nm; **Zeta Potential:** **38.25 ±** 1.06 mV	Investigate whether oxidative stress was involved in ZnO NPs-induced apoptosis and autophagy of mouse Leydig cells, and to determine the role of autophagy in ZnO NPs-induced apoptosis.	Leydig cell (Mouse)	0, 2, 3, 4, and 8	—Cell Viability —Bax, Bcl-2, cleaved Caspase-3, cleaved Caspase-8, LC3-I, LC3-II, Atg5, and Beclin 1 protein level from testis —Cell biochemistry: SOD, GPx, MDA, GSH	—Cell viability inhibition and apoptosis induction by oxidative stress; —Autophagy plays a cytoprotective role in ZnO NPs-induced	[75]

**Table 2:** In Vivo studies of ZnO NPs effects on the male reproductive system and male fertility. Abbreviations: ZnO NPs, zinc oxide nanoparticles; CAT, catalase; CM, crystal morphology; HS, hydrodynamic size; MDA, malondialdehyde; SOD, superoxide dismutase; GPx, glutathione peroxidase; GRD, glutathione reductase; GSH, reduced glutathione; GST, glutathione S-transferase; TAC, total antioxidant capacity; GSI, gonadosomatic index; Ip, intraperitoneal; SA, surface area; SE, seminiferous epithelium, STD, seminiferous tubule diameter; GET, germinal epithelium thickness; 3β-HSD, 3β-hydroxysteroid dehydrogenase; 17β-HSD, 17β-hydroxysteroid dehydrogenases; Nr5A1, steroidogenic factor 1 (Nr5A1); Mrna, messenger ribonucleic acid; ROS, reactive oxygen species; MnPCEs, micronucleated polychromatic erythrocytes; DNA, deoxyribonucleic acid; TNF-α, tumor necrosis factor alpha; IL-4, interleukin 4; LC3, microtubule-associated protein 1A/1B-light chain 3; Atg5, autophagy related 5; BIP, immunoglobulin-binding protein; XBP1s, X-box-binding protein 1 splicing; IRE1α, ER stress-associated gene inositol-requiring protein 1α; JNK, jun kinase; C/EBP, CCAAT/enhancer binding protein; CHOP, C/EBP homologous protein; Bcl-2, B cell lymphoma-2; Bax, Bcl-2-associated X protein; StAR, steroidogenic acute regulatory protein.

ZnO NPs Characteristics	Objective	Animal Model/Tissue or Organ of Study	Administration via of Exposure	Evaluated Parameters	ZnO NPs Concentration	Results	Reference
	Evaluate the effects of ZnO NPs on spermatogenesis	NMRI mice Semen Testis	Oral	—Epididymal sperm; —Testicular histology; —SE morphometry	0, 5, 50, and 300 mg/kg	—Cytotoxicity in testicular germ cells in a dose-dependent manner (≥50 mg/kg): testis histological alterations; —Increased sperm abnormalities; —Reduction of sperm and Leydig cells number	[76]
**Size**: 10–30 nm; **SA**: 20/30 m^2^/g; **Colour**: milky white; **Crystal phase**: single; **CM**: nearly spherical; **Density**: 5.606 g/cm^3^ **Purity**: ≥99%	Investigate the effects of ZnO NPs on adult male Wistar rats	Wistar rats Epididymis Sperm Blood	Ip	—Epididymal sperm; —Serum biochemistry: SOD, GPx, MDA, TOS, TAC	0, 50, 100, 150, and 200 mg/kg	—Viability and sperm number decrease (≥50 mg/kg); —Poor sperm quality (≥100 mg/kg); —Antioxidant capacity decrease (200 mg/kg)	[64]
**Size**: 20 nm **SA**: > 90 m^2^/g **Colour**: white **CM**: Nearly spherical **Purity**: ≥99%	Investigate the effects of ZnO NPs at different doses on testis of adult mice	NMRI male mice Testis	Ip	—Testicular histology	0, 250, 500, and 700 mg/kg daily	—Testis histological alterations; —Reduction and degeneration of spermatogonia, primary spermatocyte, spermatid, sperm, and Leydig cells; —Spermatogenesis arrest	[77]
**Size**: 70 nm **Shape**: spherical **Nature**: crystalline **Dispersion**: polydisperse **Surface roughness**: high (22.9 nm)	Investigate the toxicity of ZnO NPs in testicular cells	Cd1 mice (21 day old) Epididymis Sperm Testis	Intravenous	—Sperm morphology; —SE morphometry	0, 1, and 5 mg/kg	Spermatogenesis damage by alteration of SE inducing sperm abnormalities (≥5 mg/kg, ≥49 days)	[71]
**Size**: <50 nm; **SA**: >10.8 m^2^/g; **Purity**: >97%;	Evaluate the toxicological effect of ZnO NPs on male fertility and the amelioration with querectin in Wistar Han rats.	Wistar Han rats Epididymis Sperm Testis	Intragastric intubation	—Testis biochemistry: MDA, CAT, SOD, GPx, GSH; —Epididymal sperm; —Testicular histology; —Serum testosterone level; 3β-HSD; 17β-HSD; Nr5A1 mRNA levels	0, 100, and 400 mg/kg	—Decrease of sperm live cell and Leydig cell number; —Serum testosterone level decrease and increase of abnormal sperm —Atrophy, and necrosis of ST in a dose-dependent manner; —Antioxidant capacity decrease and oxidative stress increase	[78]
**Size**: 17.9 ± 7–3 nm **Distribution range**: 1–55 nm **HS**: 721 ± 109.5 nm **Purity**: ≈100% **Surface area**: 15–25 g/m^2;^	Unravel the effects of ZnO NPs exposure on germ cell apoptosis and apoptosis-related gene expressions	*Caenorhabditis elegans* Egg	Mix of ZnO NPs or ZnCl_2_ into nematode growth medium (NGM) agar	—Apoptosis genes expression: ced-13, ced-3, ced-4, ced-9, cep-1, dpl-1, efl-1, efl-2, egl-1, egl-38, lin-35, pax-2, and sir-2.1.	0, 6.14 X10^−1^, 61.4, and 614 µM	Apoptosis of germ cells (≥61,4 µM) by upregulation of apoptosis genes (cep-1, cep-13, efl-2, egl-1, lin-35, and sir-2.1 (≥614 µM). Enhanced apoptotic effects were not fully attributed to ionic Zn, ZnO NPs also have the capacity to affect apoptotic machinery	[79]
**Size**: 80 nm	Investigate side effects of various doses of ZnO NPs on the reproductive system of albino mice	Adult albino mice Testis Prostate Seminal vesicles Epididymis	Oral	—Male reproductive system histology	0, 150, and 350 mg/kg	—Cytotoxicity of testicular tissue in a dose-dependent manner; —Damages in all tissues of reproductive system (testis, seminal vesicles, prostate, and epididymis (350 mg/kg))	[80]
**Size**: 39.45 +19.88 nm **HS**: 447.5 nm **Shape**: hexagonal **Aggregation**: large and irregular **Polydispersity** index: 0.13 nm **Zeta Potential**: −32.1 mV	Evaluate the genotoxic effect of ZnO NPs in Swiss mice	Swiss male mice Semen Liver Bone Marrow	Oral	—Semen; —Genotoxicity in blood samples: CA, MnPCEs, DNA damage; —ROS levels in liver	300–2000 mg/kg	—Genotoxic effects in a dose-dependent manner by ROS generation (2000 mg/kg): leading to genomic integrity and anomalies in spermatogenesis; —Chromosomal alterations and generation of micronucleus in bone marrow cells of male mice.	[81]
**Size**: 10–30 nm	Investigate the effect of ZnO NPs on some of the antioxidant parameters of semen plasma, quantitative and qualitative properties of Arabic ram sperm	Arabic sheep Semen	Oral	—Semen; —Membrane integrity; —SOD and TAC	0, 40, and 80 mg/kg	Improves the qualitative and quantitative properties of sperm and some antioxidant parameters of seminal plasma, neutralizing the ROS effects (80 mg/kg).	[82]
**Size**: 10–30 nm **Purity**: 99.9%	Investigate the effects of different zinc source (nano, organic, and inorganic) supplementations on the reproduction of male Japanese quail.	Japanese quail chick (one-day old) Testis Eggs	Oral	—Index of cloacal gland size; —GSI; STD; GET; —Serum testosterone level; —Fertility; —Hatchability	0, 25, and 50 mg/kg	Detrimental effects on reproduction, by reducing hatchability and, also, inducing abnormalities in Japanese quail embryos	[83]
**Size**: 50 nm; **Shape**: cube; **Colour**: White **Purity**: 99.99%;	Evaluate the effects of ZnO NPs on the weight of epididymis, testis, seminal vesicle, and prostate and identify abnormalities of epididymis sperm of albino rats	NMRI male mice Testis Seminal Vesicles Prostate Sperm Epididymis	Oral	—Testis, epididymis, seminal vesicle, and prostate histology; —Testis, epididymis, seminal vesicle, and prostate weights	0, 100, and 200 mg/kg	—Testicular and epididymis weight decrease and hypertrophy of seminal vesicle and prostate; —Increase of epididymal sperm abnormalities	[84]
**Size**: <100 nm; **Purity**: ≥99.5% **Colour**: white	Detect the effects of ZnO NPs on the testes and prostate of adult albino rats and recovery	Male albino rats Serum Testis Prostate	Oral	—Serum biochemistry: MDA, GSH, CAT, SOD; —Testicular and prostatic cytokines: TNF-α, IL-4; —DNA fragmentation; —Testis and prostate histology	0 and 422 mg/kg	—Increase of oxidative stress and decrease of antioxidant capacity in serum; —Inflammatory response and DNA fragmentation increase, in testis and prostate and histological changes; —Limited exposure to ZnO NPs allows recovery of damaged tissue	[85]
**Size**: 30 nm; **HS**: 66.36 ± 0.39 nm; **Zeta Potential**: 38.25 ± 1.06 mV	Investigate whether oxidative stress was involved in ZnO NPs-induced apoptosis and autophagy of mouse Leydig cells, and to determine the role of Autophagy in ZnO NPs-induced apoptosis.	Male Kunming mice Serum Epididymis Sperm Testis	Intragastric	—Serum testosterone level; —Testis and epididymis histology; —Bax, cleaved Caspase-3, cleaved Caspase-8, LC3-I, LC3-II, Atg5, and Beclin 1 protein level from testis	0, 100, 200, and 400 mg/kg	—Disruption and atrophy of the SE by apoptosis and germ cell depletion; —Decrease of epididymal sperm density and serum testosterone level; —Induction of autophagy in testis tissue	[75]
**Size**: 30 nm; **Shape**: spherical	Evaluate the potential reproductive risks in males exposed by gavage to various doses of ZnO NPs	Male Kunming mice Serum Epididymis Sperm Testis	Ip	—Serum testosterone level; —Zinc accumulation; —Testis histology —Gene expression in Testis: BIP, XBP1s, IRE1α, JNK, C/EBP, CHOP, Bax, Bcl-2, Caspase 12, Caspase 13, StAR, P450sc	0, 50, 150, and 450 mg/kg	—Accumulation of nanoparticles contributed to seminiferous tubules degeneration and sperm cell number diminution via apoptosis and ER-stress signalling pathway; —Decrease testosterone production through the downregulation of StAR.	[86]
